# Performance and Clinical Utility of the Memorial Sloan Kettering Cancer Center Nomogram for Predicting Lymph Node Invasion in Patients Undergoing Radical Prostatectomy

**DOI:** 10.7759/cureus.116333

**Published:** 2026-09-15

**Authors:** Ana Marta Ferreira, Diogo Vidal Silva, Andre Rego, Roberto Jarimba, Juliana Santos, Pedro Nunes, Arnaldo Figueiredo

**Affiliations:** 1 Department of Urology and Renal Transplantation, Unidade Local de Saúde de Coimbra, Coimbra, PRT; 2 Urology, Faculdade de Medicina da Universidade de Coimbra, Coimbra, PRT

**Keywords:** lymph node invasion, mskcc, nomogram, pelvic lymph node dissection, prostate cancer, radical prostatectomy, risk prediction

## Abstract

Introduction

Pelvic lymph node dissection remains the most accurate method for nodal staging in patients with prostate cancer undergoing radical prostatectomy (RP), although its potential morbidity warrants careful patient selection. Preoperative nomograms may help estimate the probability of lymph node invasion (LNI) and identify patients for whom pelvic lymph node dissection could potentially be omitted. This study aimed to evaluate the discriminatory performance and potential clinical utility of the Memorial Sloan Kettering Cancer Center (MSKCC) pre-RP nomogram for predicting LNI in a contemporary, single-center cohort.

Materials and Methods

We conducted a retrospective observational study of 302 consecutive patients who underwent RP for prostate cancer between January 2020 and December 2023 at a tertiary urology center. Of these, 260 patients underwent pelvic lymph node dissection and were included in the cohort for evaluating nodal outcomes. Nomogram performance was assessed using receiver operating characteristic (ROC) curve analysis, calibration measures, and diagnostic performance at different risk thresholds. Sensitivity, specificity, positive predictive value (PPV), negative predictive value (NPV), and the proportion of potentially avoidable lymph node dissections were evaluated, with particular emphasis on an 8% predicted-risk threshold.

Results

Among the 260 patients who underwent pelvic lymph node dissection, 34/260 (13.1%) had pathologically confirmed LNI. The nomogram demonstrated good discrimination for LNI, with an area under the ROC curve (AUC) of 0.859 (95% confidence interval (CI): 0.798-0.911). At an 8% threshold, sensitivity was 34/34 (100.0%), specificity was 93/226 (41.2%), PPV was 34/167 (20.4%), and NPV was 93/93 (100.0%). Overall, 93 of the 260 pelvic lymph node dissections (93/260, 35.8%) could potentially have been avoided without missing any of the 34 patients with LNI. Increasing the threshold to 9% would have increased the proportion of potentially avoidable dissections to 112/260 (43.1%), but at the cost of missing 1/34 (2.9%) patients with LNI. The mean predicted risk exceeded the observed prevalence of LNI (17.1% vs. 34/260 (13.1%)), suggesting a degree of risk overestimation.

Conclusions

The MSKCC nomogram showed good discriminatory performance for LNI in this cohort. An 8% predicted-risk threshold identified all 34 patients with pathologically confirmed LNI (34/34, 100.0%) while potentially avoiding 93/260 (35.8%) pelvic lymph node dissections. However, the model had limited specificity and demonstrated some degree of risk overestimation. Importantly, because pathological tumor stage was used in cases where clinical stage was unavailable, these results should not be considered a formal external validation of the preoperative nomogram. Further validation using exclusively preoperative variables is warranted before routine adoption of this threshold.

## Introduction

Prostate cancer is one of the most frequently diagnosed malignancies in men and encompasses a heterogeneous spectrum of disease. Accurate risk stratification remains essential for treatment selection and determining the extent of surgical staging in patients with clinically localized or locally advanced disease. Lymph node invasion (LNI) is an important adverse pathological feature in patients undergoing radical prostatectomy (RP).

Extended pelvic lymph node dissection (ePLND) remains the most accurate method for pathological nodal staging, providing information relevant to prognosis and subsequent management [1-4]. However, a clear therapeutic benefit of pelvic lymph node dissection has not been consistently demonstrated, while increasing the extent of lymph node dissection is associated with longer operative times and increased perioperative morbidity, particularly lymphocele formation and thromboembolic complications [1-4]. Current European Association of Urology (EAU) guidance defines ePLND as the removal of lymphatic tissue along the external iliac vessels, within the obturator fossa, and along the internal iliac vessels [4].

Patient selection for ePLND is therefore clinically relevant. Conventional CT and MRI have limited sensitivity for microscopic nodal metastases [5]. Prostate-specific membrane antigen PET/CT (PSMA PET/CT) has substantially improved staging accuracy, but a negative examination does not reliably exclude small-volume pelvic nodal disease. In a prospective phase III study, ^68Ga-PSMA-11 PET showed high specificity but only 40% sensitivity for pelvic nodal metastases when histopathology was the reference standard [6]. Contemporary EAU guidelines similarly acknowledge that small metastatic lymph nodes below PET spatial resolution may remain undetected [4].

Clinical prediction models consequently remain useful for estimating the individual probability of LNI. The Memorial Sloan Kettering Cancer Center (MSKCC) pre-RP nomogram integrates preoperative clinicopathological information to estimate the probability of nodal involvement [7]. Previous studies evaluating the MSKCC and Briganti models have generally demonstrated good discrimination, although calibration and the clinical consequences of applying individual risk thresholds vary across populations [8-13].

The MSKCC model is dynamic and periodically updated, emphasizing the importance of evaluating its performance in specific clinical populations [7]. More recent models incorporating MRI and PSMA PET information may further improve nodal risk prediction [13,14]. Nevertheless, established tools such as the MSKCC nomogram remain widely studied and can provide useful information regarding the trade-off between performing ePLND and potentially missing occult nodal disease [8-12]. Current evidence therefore supports assessing not only discrimination but also calibration and the clinical consequences of applying different probability thresholds.

The objective of the present study was to assess the discriminatory performance, calibration, and potential clinical utility of the MSKCC pre-RP nomogram for predicting LNI in a contemporary single-center cohort of patients undergoing RP. We also evaluated different predicted-risk thresholds based on the proportion of ePLNDs that could potentially have been avoided and the corresponding number of patients with pathologically confirmed LNI who would have been missed.

## Materials and methods

Study design and population

This retrospective observational study included patients who underwent RP for prostate cancer at the Department of Urology and Renal Transplantation, Unidade Local de Saúde de Coimbra, Coimbra, Portugal, between January 2020 and December 2023. A total of 364 patients were screened. Sixty-two were excluded because of insufficient biopsy information, previous prostate surgery, inadequate biopsy-core data, insufficient tumor-staging information, or a prostate-specific antigen (PSA) value outside the range supported by the nomogram. The final study population comprised 302 patients. Among them, 260 underwent pelvic lymph node dissection and were included in analyses requiring pathological nodal status.

Clinical and pathological data were collected retrospectively from electronic medical records and pathology reports. Variables included age, pre-biopsy PSA, tumor stage, biopsy primary and secondary Gleason patterns, biopsy Gleason score, number of positive biopsy cores, pathological tumor stage, postoperative International Society of Urological Pathology (ISUP) Grade Group, number of lymph nodes removed, and number of metastatic lymph nodes. Gleason grading was based on the original histological grading system described by Gleason and Mellinger [15], with ISUP Grade Groups defined according to the 2014 International Society of Urological Pathology Consensus Conference [16]. Pathological nodal status was classified according to the tumor-node-metastasis staging framework as pN0 in the absence of regional nodal metastases and pN1 when at least one regional metastatic lymph node was identified [17].

Pelvic lymph node dissection was performed concomitantly with open retropubic RP and included the obturator, external iliac, and internal iliac nodal regions, corresponding to an extended pelvic lymph node dissection template [4].

Nomogram assessment and outcomes

The probability of LNI was estimated using the MSKCC pre-RP nomogram [7]. Variables available for calculation included age, pre-biopsy PSA, primary and secondary biopsy Gleason patterns, tumor stage, and biopsy-core information. The MSKCC nomogram uses clinical tumor staging based on the AJCC Version 7/2010 staging system [7,17].

Clinical tumor stage was incompletely documented in the retrospective records. When unavailable, pathological tumor stage from the RP specimen was used in the original dataset to calculate the nomogram estimate. Because pathological stage is obtained after surgery, this introduced postoperative information into a prediction model intended for preoperative use. The present study was therefore considered an assessment of nomogram performance and potential clinical utility rather than a formal external validation of the original preoperative model.

Biopsy-core information was not uniformly recorded. When the proportion of positive cores was documented as a percentage rather than as an absolute number, the number of positive cores was reconstructed from the available biopsy information. The MSKCC nomogram incorporates biopsy-core information only when both positive and negative core counts are entered; therefore, the extent to which biopsy-core information contributed to every historical nomogram estimate could not be retrospectively verified [7].

The primary outcome was discrimination between pN0 and pN1 disease. Secondary outcomes included calibration and the clinical performance of different predicted-risk thresholds, particularly the 8% threshold. Threshold performance was assessed using sensitivity, specificity, positive predictive value (PPV), negative predictive value (NPV), the proportion of potentially avoidable lymph node dissections, and the number of pN1 cases that would have been missed.

Statistical analysis

Continuous variables were summarized using mean ± standard deviation (SD) or median and range, as appropriate, and categorical variables were reported using absolute frequencies and percentages. Comparisons between pN0 and pN1 groups were performed using the two-sided Mann-Whitney U test. For each comparison, the Mann-Whitney U statistic and corresponding p-value were reported. Discrimination was assessed using receiver operating characteristic (ROC) curve analysis and quantified using area under the ROC curve (AUC) with a 95% confidence interval (CI). The 95% CI for the AUC was estimated using 10,000 bootstrap resamples. Calibration was evaluated by comparing predicted and observed probabilities of LNI and by calculating the Brier score, calibration intercept, calibration slope, and calibration-in-the-large. Discrimination, calibration, and overall predictive performance were interpreted according to established methods for evaluating clinical prediction models [18].

Diagnostic performance was assessed across different predicted-risk thresholds. A patient was considered potentially eligible for omission of pelvic lymph node dissection when the predicted risk was below the selected threshold. A two-sided p-value < 0.05 was considered statistically significant. The original descriptive analyses were performed using IBM SPSS Statistics version 30.0 (IBM Corp., Armonk, NY). Additional ROC, bootstrap, calibration, threshold, and Mann-Whitney U analyses were recalculated from the final database using Python version 3.13.5 with NumPy version 2.3.5, SciPy version 1.17.0, scikit-learn version 1.8.0, and statsmodels version 0.14.6.

Ethical considerations

The study was approved by the Ethics Committee of the Faculty of Medicine of the University of Coimbra (FMUC) on February 11, 2025 (Approval No. CE-018/2025).

## Results

Study population

Of the 364 patients screened during the study period, 62 were excluded, resulting in a final cohort of 302 patients. The main clinical and biopsy characteristics of the study population are summarized in Table 1. The mean age was 67.03 ± 6.13 years, and the median pre-biopsy PSA level was 8.42 ng/mL (range: 0.80-65.00 ng/mL). The median MSKCC-predicted probability of lymph node invasion was 9% (range: 1%-83%).

**Table 1:** Clinical and biopsy characteristics of the overall study population (N = 302) SD: standard deviation; PSA: prostate-specific antigen; MSKCC: Memorial Sloan Kettering Cancer Center; LNI: lymph node invasion; ePLND: extended pelvic lymph node dissection

Variables	Values
Age, years, mean ± SD	67.03 ± 6.13
Age, years, median (range)	68 (48-79)
Pre-biopsy PSA, ng/mL, mean ± SD	11.44 ± 8.90
Pre-biopsy PSA, ng/mL, median (range)	8.42 (0.80-65.00)
Primary biopsy Gleason pattern, mean ± SD	3.30 ± 0.47
Secondary biopsy Gleason pattern, mean ± SD	3.57 ± 0.57
Biopsy Gleason score, mean ± SD	6.88 ± 0.66
Positive biopsy cores, median (range)	2 (1-11)
MSKCC-predicted LNI risk, %, mean ± SD	15.75 ± 16.62
MSKCC-predicted LNI risk, %, median (range)	9 (1-83)
Underwent ePLND, n/N (%)	260/302 (86.1%)
Pathologically confirmed pN1 among ePLND patients, n/N (%)	34/260 (13.1%)

Among the 302 included patients, 260/302 (86.1%) underwent pelvic lymph node dissection and were therefore available for pathological nodal assessment. Of these, 34/260 (13.1%) had pathologically confirmed LNI (pN1), whereas 226/260 (86.9%) had no lymph node involvement (pN0). Patients with pN1 disease showed a less favorable clinicopathological profile than those with pN0 disease (Table 2). The primary biopsy Gleason pattern was significantly higher in the pN1 group (3.79 ± 0.48 vs. 3.27 ± 0.44; Mann-Whitney U = 1910.5, p < 0.001), as was the total biopsy Gleason score (7.44 ± 0.75 vs. 6.88 ± 0.59; Mann-Whitney U = 2359.5, p < 0.001). The number of positive biopsy cores was also significantly greater among patients with pN1 disease, with a median of five positive cores compared with two in the pN0 group (Mann-Whitney U = 1564.0, p < 0.001).

**Table 2:** Comparison of clinical and pathological variables according to nodal status Between-group comparisons were performed using the two-sided Mann-Whitney U test. The U statistic and corresponding p-value are reported for each comparison. Mean ± SD and median (range) are both provided for selected continuous variables for descriptive purposes; a single Mann-Whitney U test was performed for each variable pN0: no pathological lymph node invasion; pN1: pathological lymph node invasion; SD: standard deviation; PSA: prostate-specific antigen; MSKCC: Memorial Sloan Kettering Cancer Center

Variables	pN0 (n = 226)	pN1 (n = 34)	Mann-Whitney U	P-value
Age, years, mean ± SD	66.98 ± 6.24	67.85 ± 5.67	3523.0	0.435
Age, median (range)	68 (51-79)	68.5 (55-75)		
PSA, ng/mL, mean ± SD	11.41 ± 8.93	13.54 ± 9.06	3244.5	0.144
PSA, median (range)	8.90 (0.80-65.00)	9.82 (3.63-34.00)		
Primary biopsy Gleason pattern, mean ± SD	3.27 ± 0.44	3.79 ± 0.48	1910.5	< 0.001
Secondary biopsy Gleason pattern, mean ± SD	3.61 ± 0.56	3.65 ± 0.69	3815.0	0.941
Total biopsy Gleason score, mean ± SD	6.88 ± 0.59	7.44 ± 0.75	2359.5	< 0.001
Positive biopsy cores, median (range)	2 (1-11)	5 (1-11)	1564.0	< 0.001
MSKCC-predicted risk, %, mean ± SD	14.09 ± 14.16	36.76 ± 18.13	1087.0	< 0.001
MSKCC-predicted risk, %, median (range)	9 (1-73)	36.5 (8-73)		
Lymph nodes removed, mean ± SD	10.23 ± 5.02	10.35 ± 4.30	3666.0	0.667
Lymph nodes removed, median (range)	9 (1-35)	9 (2-24)		

The MSKCC-predicted probability of LNI differed markedly according to pathological nodal status. Patients with pN1 disease had a mean predicted risk of 36.76 ± 18.13%, compared with 14.09 ± 14.16% in patients with pN0 disease. The median predicted risk was 36.5% in the pN1 group and 9% in the pN0 group (Mann-Whitney U = 1087.0, p < 0.001). By contrast, no statistically significant differences were observed between pN0 and pN1 patients for age (Mann-Whitney U = 3523.0, p = 0.435), pre-biopsy PSA (U = 3244.5, p = 0.144), secondary biopsy Gleason pattern (U = 3815.0, p = 0.941), or the number of lymph nodes removed (U = 3666.0, p = 0.667). The median number of lymph nodes removed was nine in both groups.

Discriminatory performance of the MSKCC nomogram

ROC analysis showed that the MSKCC nomogram had good discriminatory performance for pathological lymph node invasion. The AUC was 0.859 (95% CI: 0.798-0.911) (Figure 1), indicating good discrimination between patients with pN0 and pN1 disease.

**Figure 1:**
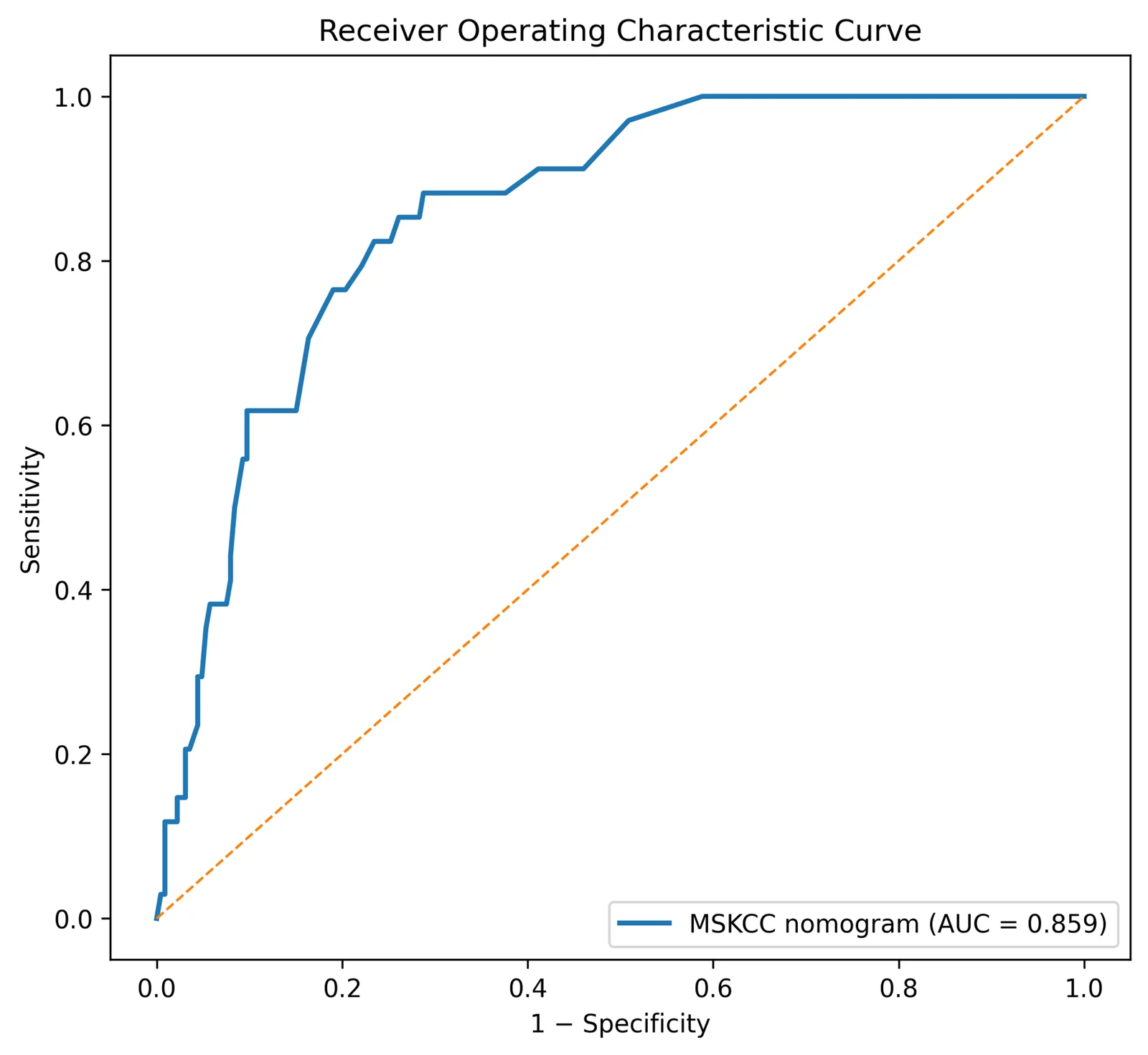
Receiver operating characteristic curve of the MSKCC nomogram for pathological lymph node invasion The receiver operating characteristic curve demonstrates the discriminatory ability of the MSKCC-predicted probability for distinguishing pN1 from pN0 disease. The AUC was 0.859 (95% CI: 0.798-0.911) MSKCC: Memorial Sloan Kettering Cancer Center; AUC: area under the curve; CI: confidence interval

The distribution of predicted probabilities was consistent with this finding, as patients with pathologically confirmed LNI had substantially higher nomogram estimates than patients without nodal disease.

Performance at different predicted-risk thresholds

The clinical consequences of applying different MSKCC risk thresholds are summarized in Table 3.

**Table 3:** Clinical performance of different MSKCC risk thresholds Sensitivity, specificity, PPV, and NPV are presented as the corresponding numerator/denominator and percentage MSKCC: Memorial Sloan Kettering Cancer Center; ePLND: extended pelvic lymph node dissection; PPV: positive predictive value; NPV: negative predictive value; pN1: pathological lymph node invasion

Threshold for ePLND	Sensitivity, n/N (%)	Specificity, n/N (%)	PPV, n/N (%)	NPV, n/N (%)	Potentially avoided ePLNDs, n/N (%)	Missed pN1 cases, n/N (%)
≥ 5%	34/34 (100.0%)	52/226 (23.0%)	34/208 (16.3%)	52/52 (100.0%)	52/260 (20.0%)	0/34 (0.0%)
≥ 6%	34/34 (100.0%)	67/226 (29.6%)	34/193 (17.6%)	67/67 (100.0%)	67/260 (25.8%)	0/34 (0.0%)
≥ 7%	34/34 (100.0%)	82/226 (36.3%)	34/178 (19.1%)	82/82 (100.0%)	82/260 (31.5%)	0/34 (0.0%)
≥ 8%	34/34 (100.0%)	93/226 (41.2%)	34/167 (20.4%)	93/93 (100.0%)	93/260 (35.8%)	0/34 (0.0%)
≥ 9%	33/34 (97.1%)	111/226 (49.1%)	33/148 (22.3%)	111/112 (99.1%)	112/260 (43.1%)	1/34 (2.9%)
≥ 10%	31/34 (91.2%)	122/226 (54.0%)	31/135 (23.0%)	122/125 (97.6%)	125/260 (48.1%)	3/34 (8.8%)
≥ 15%	30/34 (88.2%)	157/226 (69.5%)	30/99 (30.3%)	157/161 (97.5%)	161/260 (61.9%)	4/34 (11.8%)
≥ 20%	28/34 (82.4%)	171/226 (75.7%)	28/83 (33.7%)	171/177 (96.6%)	177/260 (68.1%)	6/34 (17.6%)

At a threshold of 8%, all 34/34 patients with pN1 disease (100.0%) were classified above the threshold. Specificity was 93/226 (41.2%), PPV was 34/167 (20.4%), and NPV was 93/93 (100.0%). At this threshold, 93 of the 260 patients undergoing pelvic lymph node dissection had an MSKCC-predicted risk below 8%. Therefore, 93/260 lymph node dissections (35.8%) could potentially have been avoided without missing any of the 34 patients with pN1 disease in this cohort.

Lower thresholds preserved a sensitivity of 34/34 (100.0%) but resulted in fewer potentially avoidable procedures. At thresholds of 5%, 6%, and 7%, 52/260 (20.0%), 67/260 (25.8%), and 82/260 (31.5%) pelvic lymph node dissections, respectively, could potentially have been avoided. Conversely, increasing the threshold above 8% increased the number of potentially avoided lymph node dissections but resulted in missed cases of pathological nodal disease. At a threshold of 9%, 112/260 procedures (43.1%) could potentially have been avoided, but 1/34 pN1 patients (2.9%) would have been missed. At a threshold of 10%, 125/260 procedures (48.1%) could potentially have been avoided, at the cost of missing 3/34 pN1 cases (8.8%).

At higher thresholds, this trade-off became more pronounced. A 15% threshold would potentially have avoided 161/260 lymph node dissections (61.9%) while missing 4/34 pN1 patients (11.8%), whereas a 20% threshold would potentially have avoided 177/260 procedures (68.1%) but would have missed 6/34 patients with lymph node invasion (17.6%). Thus, within this cohort, 8% represented the highest integer threshold at which no patient with pathologically confirmed lymph node invasion was missed.

Calibration

Calibration analysis demonstrated that the mean MSKCC-predicted probability of LNI was 17.1%, whereas the observed prevalence of pN1 disease was 34/260 (13.1%), suggesting a tendency toward overall risk overestimation. The Brier score was 0.091. The calibration intercept was -0.137, the calibration slope was 1.236, and calibration-in-the-large was -0.382. The relationship between predicted and observed probabilities across risk strata is shown in Figure 2.

**Figure 2:**
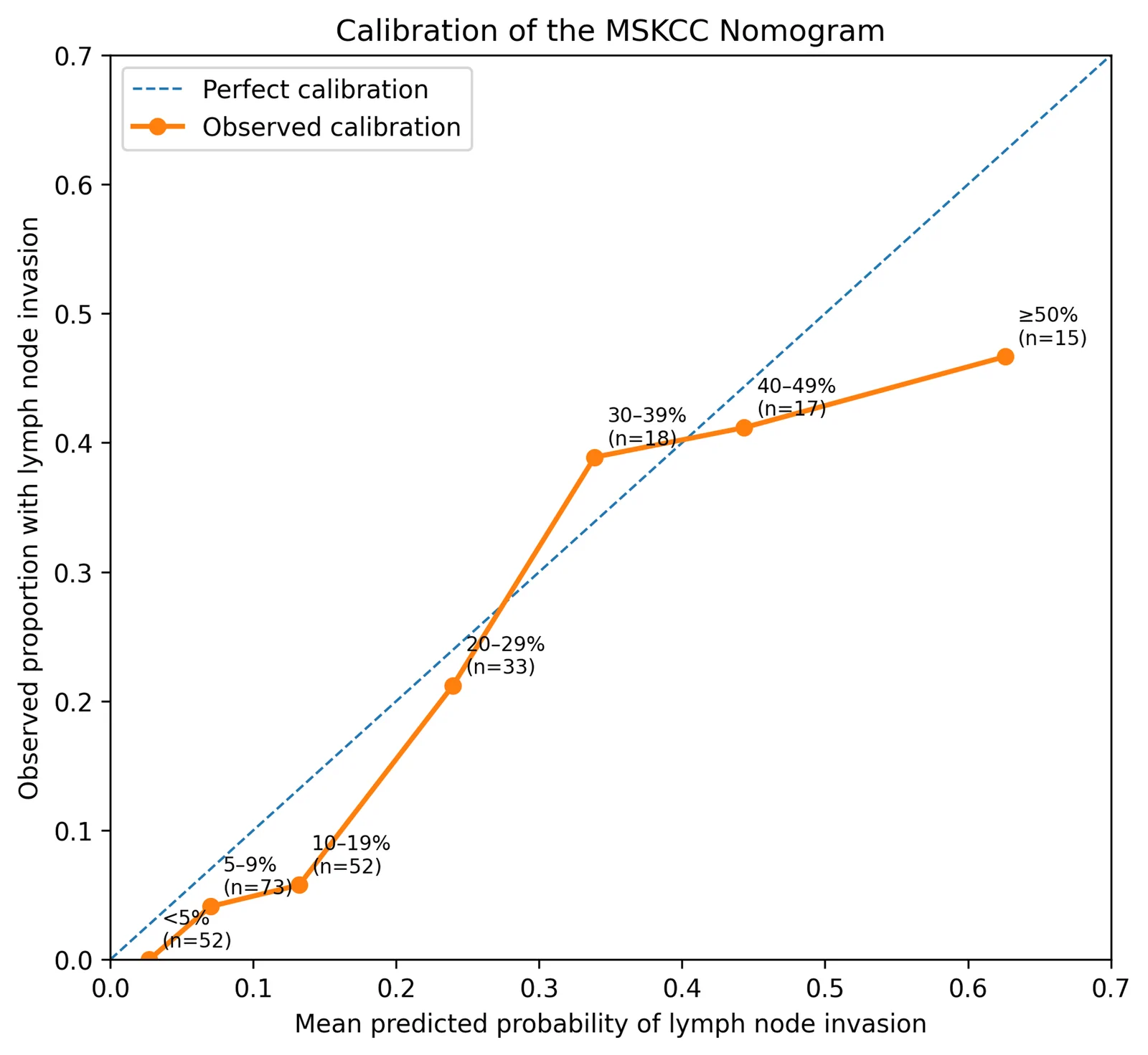
Calibration of MSKCC-predicted and observed lymph node invasion risk The dashed diagonal line represents perfect calibration. The plotted points represent observed lymph node invasion rates across increasing categories of MSKCC-predicted risk MSKCC: Memorial Sloan Kettering Cancer Center

The discrepancy between predicted and observed risk was particularly apparent in the lower and intermediate risk categories. Among patients with a predicted risk below 5%, 0/52 (0.0%) had LNI. Among patients with predicted risks of 5%-9%, 3/73 (4.1%) had pN1 disease, while 3/52 (5.8%) patients in the 10%-19% predicted-risk group had pN1 disease. At higher predicted probabilities, observed LNI rates increased progressively: 7/33 (21.2%) in the 20%-29% group, 7/18 (38.9%) in the 30%-39% group, 7/17 (41.2%) in the 40%-49% group, and 7/15 (46.7%) among patients with predicted risks of 50% or greater. Overall, these findings indicate that the nomogram demonstrated good discrimination but tended to overestimate the absolute risk of LNI in several risk strata.

## Discussion

In this retrospective single-center study, the MSKCC pre-RP nomogram showed good discriminatory performance for pathological LNI, with an AUC of 0.859. The most clinically relevant finding was that an 8% predicted-risk threshold identified all 34/34 patients with pN1 disease (100.0%) while potentially allowing the omission of pelvic lymph node dissection in 93/260 patients (35.8%). These findings are consistent with previous evaluations of the MSKCC and other established nomograms, which have generally demonstrated good discrimination but variable performance across populations [8-12]. Differences in case mix, disease prevalence, staging methods, and threshold selection may explain this variability and reinforce the need to assess the clinical consequences of specific cutoffs rather than relying on discrimination alone.

In the present cohort, an 8% threshold provided the highest integer cutoff at which no pN1 case was missed. Lower thresholds preserved a sensitivity of 34/34 (100.0%) but avoided fewer procedures, whereas thresholds above 8% progressively increased the proportion of potentially avoidable lymph node dissections at the cost of missed nodal disease. This trade-off is clinically relevant because the primary role of the nomogram is not to diagnose pN1 disease, but to identify patients with sufficiently low predicted risk for whom pelvic lymph node dissection might potentially be omitted. Similar threshold-dependent trade-offs have been reported in previous external validation studies [8-12].

At the 8% threshold, the NPV was 93/93 (100.0%), and none of the patients with a predicted risk below this value had pathologically confirmed LNI. Nevertheless, this finding should not be interpreted as evidence that 8% represents a universally optimal threshold. The clinical consequences of any cutoff depend on disease prevalence, case mix, biopsy characteristics, staging practices, and model calibration. Previous studies have proposed different thresholds for the MSKCC and Briganti nomograms, reflecting variation between populations [8-12]. The present analysis should therefore be considered an assessment of threshold performance within this specific cohort rather than a recommendation to replace established guideline-based decision-making.

Calibration analysis provided additional information beyond discrimination. Although the nomogram correctly ranked patients according to risk, the mean predicted probability of LNI was 17.1%, compared with an observed pN1 prevalence of 34/260 (13.1%), suggesting a degree of overall risk overestimation. This distinction is important because a prediction model may discriminate well while still providing imperfect estimates of absolute risk, particularly when treatment decisions are based on numerical thresholds [18]. The role of nomograms should also be interpreted in the context of contemporary staging. PSMA PET/CT has improved the detection of nodal and distant metastatic disease, but small-volume nodal metastases may remain undetected [4,6]. Prediction models may therefore continue to provide complementary information, particularly when estimating the probability of occult nodal disease and selecting patients for pelvic lymph node dissection [13,14].

Several limitations of this study should be acknowledged. The retrospective and single-center design may introduce selection bias and limit generalizability. In addition, pathological nodal status was available only in patients who underwent lymph node dissection, and only 34 patients had pN1 disease, which limits the precision of threshold-specific estimates. The main methodological limitation concerns tumor staging. Clinical T-stage was incompletely documented, and pathological T-stage from the RP specimen was used when clinical stage was unavailable. This introduces postoperative information into a model designed for preoperative use and may have influenced the apparent performance of the nomogram.

Accordingly, the present study should be regarded as an assessment of model performance and potential clinical utility rather than a formal external validation. Biopsy-core information was also inconsistently documented and required reconstruction in some patients. Because the MSKCC nomogram only incorporates core information when both positive and negative core counts are available, the contribution of biopsy-core data to all historical estimates could not be confirmed retrospectively [7].

Despite these limitations, the study provides a clinically interpretable assessment of the MSKCC nomogram by combining discrimination, calibration, and threshold-specific consequences. Future multicenter studies using exclusively preoperative variables and contemporary imaging should determine whether an 8% threshold offers an appropriate balance between avoiding unnecessary lymph node dissection and minimizing the risk of missed nodal disease.

## Conclusions

The MSKCC nomogram demonstrated good discriminatory performance for pathological LNI in this cohort. At an 8% predicted-risk threshold, all 34/34 patients with pN1 disease (100.0%) were identified, while 93/260 pelvic lymph node dissections (35.8%) could potentially have been avoided. However, the model showed limited specificity and some degree of overestimation of absolute LNI risk. Because pathological tumor stage was used when clinical stage was unavailable, these findings should not be considered a formal external validation. Further studies using exclusively preoperative variables are required before routine adoption of an 8% threshold can be recommended.
